# Prognostic value of early radiological response to first‐line platinum‐containing chemotherapy in patients with metastatic nasopharyngeal carcinoma

**DOI:** 10.1002/cam4.2751

**Published:** 2019-12-13

**Authors:** Guo‐Ying Liu, Wang‐Zhong Li, Kang‐Qiang Peng, Xing Lv, Liang‐Ru Ke, Yi‐Shan Wu, De‐Ling Wang, Hu Liang, Kui‐Yuan Liu, Shu‐Hui Lv, Xiang Guo, Yan‐Qun Xiang, Wei‐Xiong Xia

**Affiliations:** ^1^ State Key Laboratory of Oncology in South China and Collaborative Innovation Center for Cancer Medicine Sun Yat‐Sen University Cancer Center Guangzhou China; ^2^ Department of Nasopharyngeal Carcinoma Sun Yat‐Sen University Cancer Center Guangzhou China; ^3^ Imaging Diagnosis and Interventional Center Sun Yat‐Sen University Cancer Center Guangzhou China

**Keywords:** early radiological response, first‐line chemotherapy, metastatic nasopharyngeal carcinoma

## Abstract

**Background:**

To explore the prognostic value of early radiological response (ERR) to first‐line platinum‐containing chemotherapy in patients with metastatic nasopharyngeal carcinoma (mNPC), as well as its correlation with the best radiological response (BRR).

**Patients and methods:**

A total of 756 mNPC patients with measurable lesions who received first‐line platinum‐containing chemotherapy were enrolled in this study. ERR was defined as complete or partial response after 6 weeks of chemotherapy according to the Response Evaluation Criteria in Solid Tumors (RECIST) 1.1. We performed survival analyses according to the radiological response after repeated chemotherapy. Log‐rank test and Cox regression were used to analyze the survival data.

**Results:**

About 470 patients achieved ERR and 78 patients achieved subsequent response (objective response after repeated chemotherapy). ERR patients had better OS (*P* < .001, median OS: 34.3 vs 22.2 months) and PFS (*P* < .001, median PFS: 10.2 vs 7.4 months) than non‐ERR ones. ERR (OS: HR = 0.591, 95% CI, 0.495‐0.705, *P* < .001, PFS: HR = 0.586, 95% CI, 0.500‐0.686, *P* < .001) was independently prolonged survival compared with non‐ERR ones. Besides, ERR was significantly correlated with the BRR (Kappa: 0.73; Pearson: 0.74, *P* < .001), and had significantly longer OS and PFS than patients with subsequent response, respectively.

**Conclusion:**

ERR is an independent prognostic factor in determining survival in mNPC patients received first‐line platinum‐containing chemotherapy, which may be a more sensitive predictor to assess overall efficacy of systemic treatment than BRR in mNPC. Prospective validation studies are needed.

## Graphical abstract

The radiological response to chemotherapy is a critical feature in the clinical evaluation of antitumor effects and associated with survival outcome. Our study suggested that early radiological response is helpful in giving prognostic information for both physicians and patients, and also valuable in selecting patients for more intensive multidisciplinary treatments.

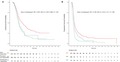

## 1 INTRODUCTION

Nasopharyngeal carcinoma (NPC), a head and neck epithelial malignancy, is highly distinct due to its epidemiology, histopathology, clinical characteristics, and methods of treatment.^1^ Despite advances in radiation and chemotherapy, more than 20% of NPC patients will develop distant metastasis after radical chemoradiotherapy.^2^ Additionally, approximately 6%‐15% newly diagnosed NPC patients have distant metastatic disease at the time of presentation.^3^ Once metastatic disease has occurred, the prognosis is generally poor with a median overall survival (OS) about 20 months.^4^, ^5^ At present, platinum‐containing chemotherapy is the standard first‐line regimen for metastatic NPC (mNPC) patients as recommended in NCCN guidelines, from which robust objective response (OR) rates have been achieved (40%‐75%).^6^

The radiological response to chemotherapy is a critical feature in the clinical evaluation of antitumor effects.^7^ Several studies have shown that a radiological response based on Response Evaluation Criteria in Solid Tumors (RECIST) predicts the survival in patients with colorectal cancer (CRC), breast cancer, soft tissue sarcoma, and renal cell carcinoma (RCC).^8^, ^9^, ^10^, ^11^ Recent studies have reported that the prognostic value of the radiological response to first‐line chemotherapy is associated with better survival for mNPC patients.

Although the importance of chemotherapy repetition in treating mNPC is now clear, there is little information on the clinical impact of the responses after each chemotherapy cycle. Defining the best time to assess response is also challenging. Compared with the best radiological response (BRR), early radiological response (ERR) has recently come to the attention of clinicians as a favorable prognostic marker for both progression‐free survival (PFS) and OS in patients with metastatic CRC and metastatic RCC while receiving first‐line treatment.^12^, ^13^, ^14^ Insufficient data have, therefore, been reported regarding the clinical relevance of ERR in mNPC patients treated with chemotherapy.

Using pooled data from a large database of mNPC treated with first‐line platinum‐containing chemotherapy, the aims of this study were to evaluate the stratified distribution and clinical implications of the ERR compared with the BRR after repeated chemotherapy.

## 2 MATERIALS AND METHODS

### 2.1 Study design and patient cohort

With institutional review board approval and a waiver of the requirement for patient consent, prospectively maintained databases were queried for all consecutive newly diagnosed mNPC patients who received full course of first‐line platinum‐containing chemotherapy at Sun Yat‐Sen University Cancer Center (SYSUCC) between September 2005 and April 2017. Patients with at least a measurable target lesion and two courses of imaging evaluation data during the first‐line chemotherapy were included. The exclusion criteria included the following: (a) other invasive malignant diseases; (b) incomplete imaging evaluation; and (c) incomplete records on clinical or follow‐up data (the collection schema is presented in Supplementary Figure 1). Additional information, including baseline data (age, gender, KPS, pathological diagnosis, clinical history), nasopharyngeal and neck magnetic resonance imaging (MRI), chest and abdomen computed tomography (CT) scan, bone scintigraphy or ^18^F‐fluorodeoxyglucose PET scan, treatment type, biochemical profile, EBV serology, and follow‐up, was collected from electronic and paper medical records.

**Figure 1:**
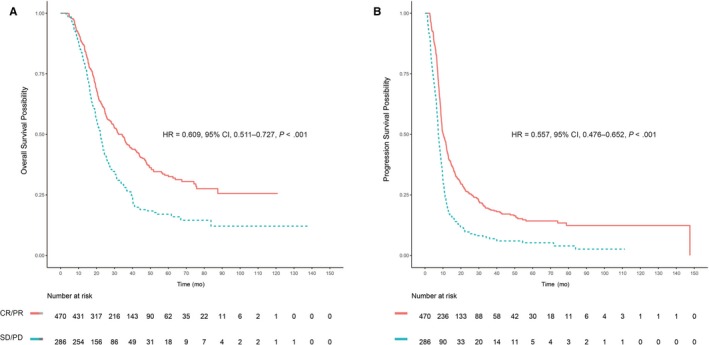
Overall survival and progression‐free survival for the patients with early radiological response. Kaplan‐Meier estimates of (A) overall survival and (B) progression‐free survival based on early radiological response to first‐line chemotherapy. Patients with a complete response (CR) or partial response (PR) were categorized as responders; those with stable disease (SD) or progressive disease (PD) were categorized as nonresponders.

ERR was defined as complete response (CR) or partial response (PR) after 6 weeks (±2 weeks)^15^ of chemotherapy (the median scan period was 6.4 weeks (range 4.8‐8.4 weeks)) and the BRR^6^ was defined as a best response of CR or PR from the initiation of treatment until disease progression or death according to the RECIST 1.1.^16^ CR was defined as the disappearance of the target lesion. PR was defined as a reduction of the sum of the longest diameter of the target lesions by at least 30%. Progressive disease (PD) was defined as an increase of the sum of the longest diameter of the target lesions by at least 20%. Response assessment by CT or MRI scans was performed every two cycles during first‐line chemotherapy and thereafter every 3 months until the disease progressed or the patient died. The imaging data were routinely analyzed by two independent radiologists to determine the treatment response. If there was a disagreement regarding the results, a team meeting was arranged to review the images side‐by‐side. If there is still disagreement after negotiation, we had to pursue the third radiologist for further evaluation (minority obeying majority).

The primary endpoint was OS, defined as the time from diagnosis of distant metastasis to death by any cause, survival was censored at last study follow‐up date. The secondary endpoint was PFS, defined as the time from the diagnosis of distant metastasis to disease progression or death by any cause.

### 2.2 Statistical analysis

Statistical analysis was performed using version 22.0 of the Statistical Package and R version 3.5.0. A Chi‐square or Fisher's exact test was used to compare categorical variables between response and nonresponse, respectively. In addition, the correlation between ERR and BRR was measured by kappa agreement and Pearson coefficient (r), and receiver operating characteristics (to assess sensitivity, specificity, NPV, PPV). Life‐table estimation was performed using the Kaplan‐Meier method and log‐rank test. The multivariate Cox proportional hazards model was used to calculate hazard ratios and 95% confidence intervals. All tests were two‐sided, and a *P* < .05 was considered significant.

## 3 RESULTS

### 3.1 Patient characteristics

The records of 1391 consecutive patients with mNPC who received treatment between September 2005 and April 2017 were retrieved from the prospectively maintained institution's database. A total of 756 mNPC patients with measurable lesions who received first‐line platinum‐containing chemotherapy were eligible for the analysis. The baseline characteristics of the patients are shown in Table 1. Among the patients, 367 patients were primary distant metastasis and 389 patients were recurrence with distant metastases. The median age was 45 years, and most of the patients were male (82.7%), had an Eastern Cooperative Oncology Group performance status (ECOG PS) of 0‐1 (92.7%), and WHO type III histology (96.3%). Three hundred and ninety‐one had more than one organ involved with metastasis (51.7%). The most frequent metastasis locations were the liver (44.4%), lung (49.2%), and bone (45.2%). The pretreatment plasma EBV DNA levels ranged from 0 to 85 000 000 000 copies/mL (median, 29 950 copies/mL). Of the 756 patients, 33.7% (n = 255) received platinum plus 5‐fluorouracil (PF) regimen, 25.9% (n = 196) of patients received platinum plus docetaxel or paclitaxel (TP) regimen, 22.4% (n = 169) of patients received triplet regimen consisting of docetaxel or paclitaxel plus cisplatin and 5‐fluorouracil (TPF), and the remaining patients (n = 136) received platinum plus gemcitabine (GP) regimen.

**Table 1:** Patient and disease characteristics

Characteristics	Entire group (%)	Radiological response					
		Early radiological response			Best radiological response		
		Responder (%)	Nonresponder (%)	*P*	Responder (%)	Nonresponder (%)	*P*
Sex				.278			.248
Male	625 (82.7)	383 (61.3)	242 (38.7)		435 (69.6)	190 (30.4)	
Female	131(17.3)	87 (66.4)	44 (33.6)		98 (74.8)	33 (25.2)	
Age				.368			.873
<45 y	356 (47.1)	215 (60.4)	141 (39.6)		250 (70.2)	106 (29.8)	
≥45 y	400 (52.9)	255 (63.8)	145 (36.2)		283 (70.8)	117 (29.2)	
ECOG PS				.565			1.000
0‐1	701 (92.7)	438 (62.5)	263 (37.5)		494 (70.5)	207 (29.5)	
>1	55 (7.3)	32 (58.2)	23 (41.8)		39 (70.9)	16 (29.1)	
Histology				.492			.811
I	1 (0.1)	1 (100)	0 (0)		1(100)	0 (0)	
II	27 (3.6)	19 (70.4)	8 (29.6)		19 (70.4)	8 (29.6)	
III	728 (96.3)	480 (65.9)	278 (34.1)		513 (70.5)	215 (29.5)	
Smoking status				.174			.630
Yes	422 (55.8)	253 (60.0)	169 (40.0)		294 (69.7)	128 (30.3)	
No	334 (44.2)	217 (65.0)	117 (35.0)		239 (71.6)	95 (28.4)	
Drinking status				.577			.177
Yes	505 (66.8)	310 (61.4)	195 (38.6)		348 (68.9)	157 (31.1)	
No	251 (33.2)	160 (63.7)	91 (36.3)		185 (73.7)	66 (26.3)	
Stage				.261			.002
Primary metastases	367 (48.5)	236 (64.3)	131 (35.7)		278 (75.7)	89 (24.3)	
Recurrence with distant metastases	389 (51.5)	234 (60.2)	155 (39.8)		255 (69.5)	134 (30.5)	
Number of metastatic organs				.072			.046
One	365 (48.3)	239 (65.5)	126 (34.5)		270 (74.0)	95 (26.0)	
Two or more	391 (51.7)	231 (59.1)	160 (40.9)		263 (67.3)	128 (22.7)	
Liver metastasis				.407			.127
Absent	420 (55.6)	267 (63.6)	153 (36.4)		306 (72.9)	114 (27.1)	
Present	336 (44.4)	203 (60.4)	133 (39.6)		227 (67.6)	109 (22.4)	
Lung metastasis				.454			.151
Absent	384 (50.8)	244 (63.5)	140 (36.5)		280 (72.9)	104 (27.1)	
Present	372 (49.2)	226 (60.8)	146 (39.2)		253 (68.0)	119 (32.0)	
Bone metastasis				.821			.810
Absent	414 (54.8)	259 (62.6)	155 (37.4)		290 (70.0)	124 (30.0)	
Present	342 (45.2)	211 (61.5)	131 (38.5)		243 (70.8)	99 (29.2)	
Chemotherapy regimen				.003			.001
PF	255 (33.7)	146 (54.9)	109 (45.1)		165 (64.7)	90 (35.3)	
TP	196 (25.9)	110 (56.1)	86 (43.9)		125 (73.8)	71 (26.2)	
TPF	169 (22.4)	119 (70.4)	50 (29.6)		137 (81.1)	32 (18.9)	
GP	136 (18.0)	95 (69.9)	41 (30.1)		106 (77.9)	30 (22.1)	
EBV DNA level				.346			.070
≤1000 copies	148 (19.6)	87 (61.7)	61 (38.3)		95 (64.2)	53 (35.8)	
>1000 copies	608 (80.4)	383 (63.0)	225 (37.0)		438 (72.0)	170 (28.0)	
LDH level				.937			.613
≤250 U/L	505 (66.8)	313 (61.9)	192 (38.1)		359 (71.1)	146 (28.9)	
>250 U/L	251 (33.2)	157 (62.5)	94 (37.5)		174 (69.3)	77 (30.7)	
ALP level				.425			.338
≤125 U/L	629 (83.2)	395 (62.8)	234 (37.2)		448 (71.2)	181 (28.8)	
>125 U/L	127 (16.8)	75 (59.1)	52 (40.9)		85 (66.9)	42 (33.1)	
CRP level				.815			.804
≤3.0 mg/L	274 (36.2)	172 (62.8)	102 (37.2)		195 (71.2)	79 (28.8)	
>3.0 mg/L	482 (63.8)	298 (61.8)	184 (38.2)		338 (70.1)	144 (29.9)	

### 3.2 ERR and clinical outcome

**Figure 2:**
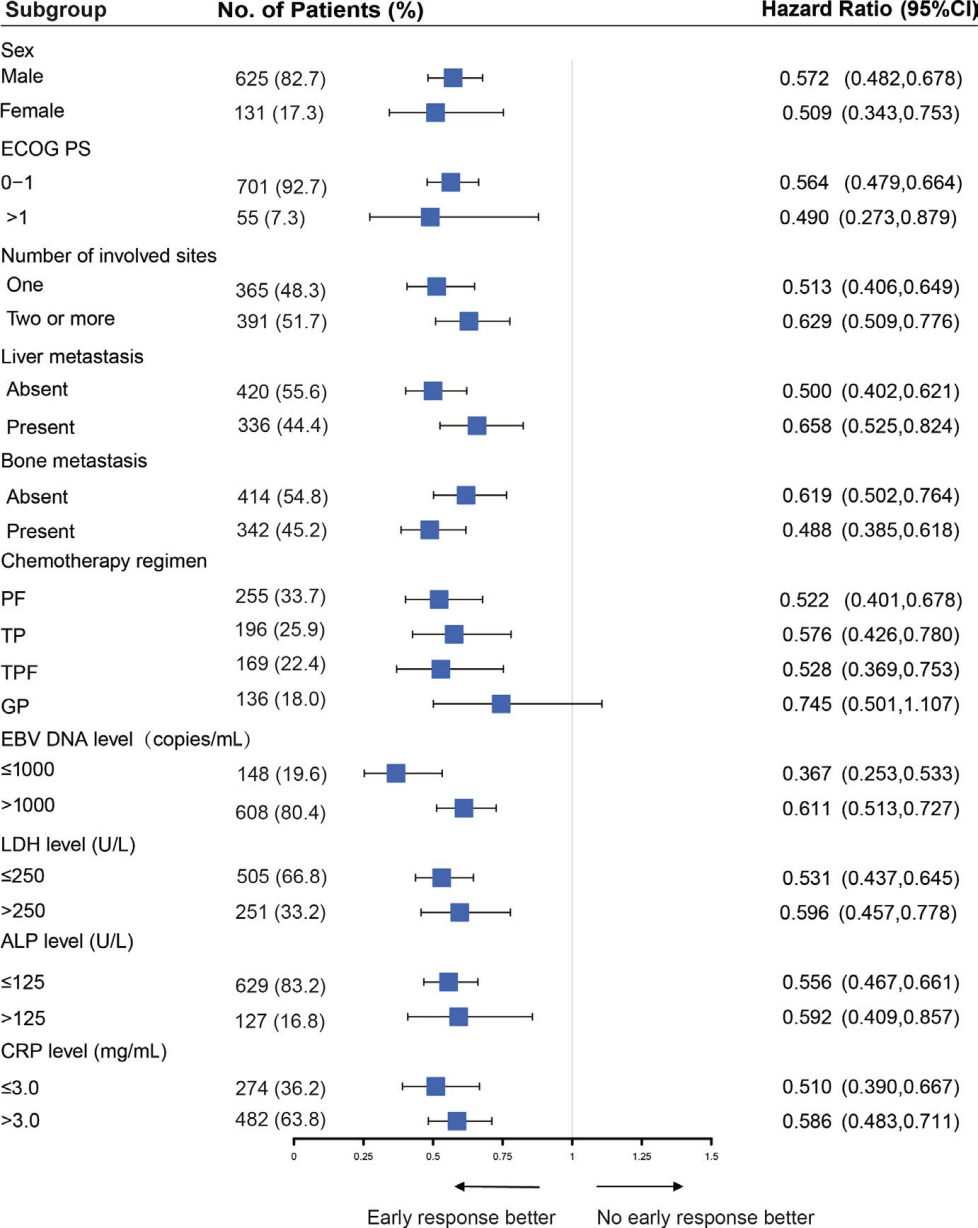
An overall survival analysis to evaluate treatment effect of early radiological responder vs nonresponder in various subgroup

**Figure 3:**
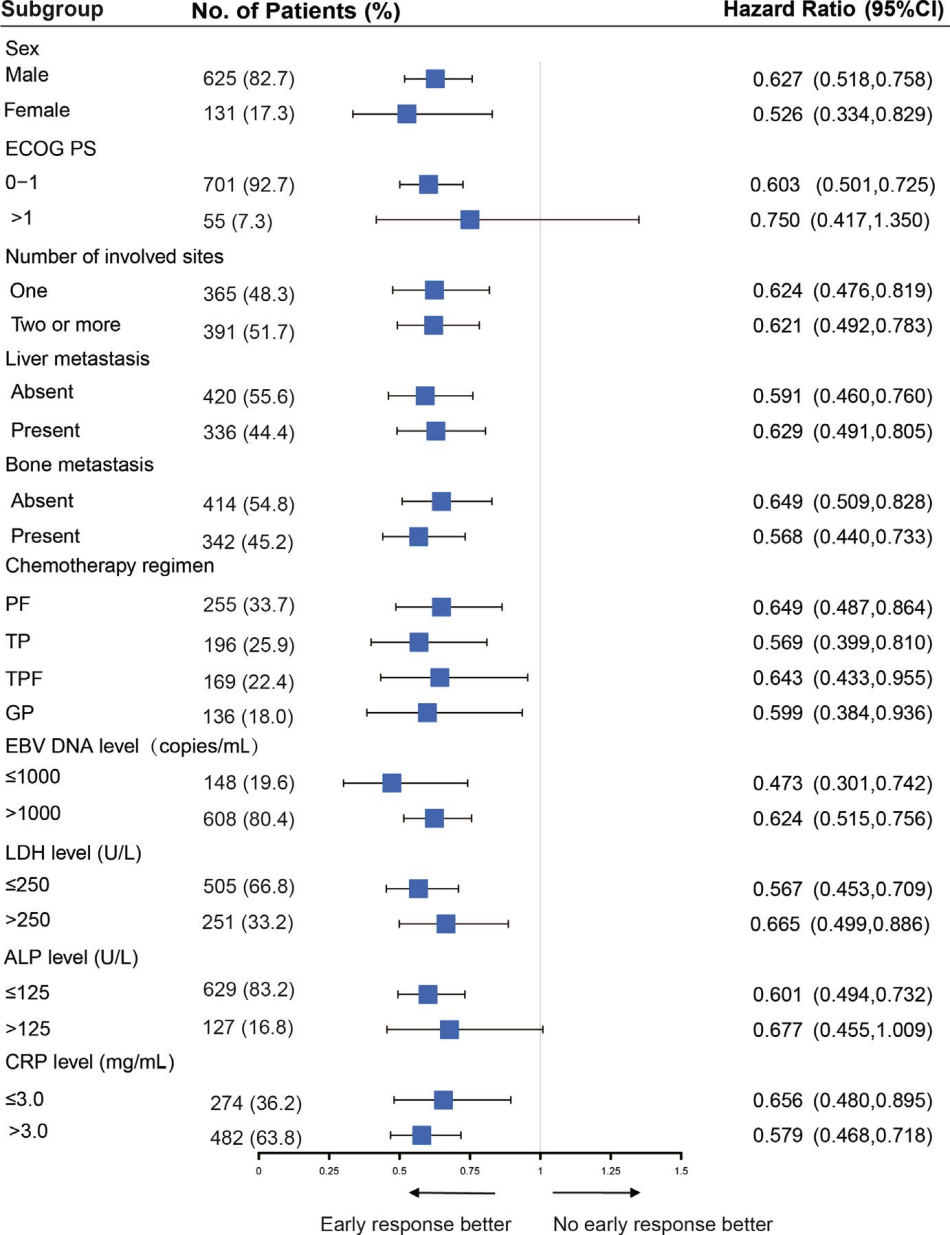
A progression‐free survival analysis to evaluate treatment effect of early radiological responders vs nonresponders in various subgroup

Among 756 patients, 470 patients achieved ERR after 6 weeks of chemotherapy and 286 patients did not. Patient demographics and baseline characteristics were generally similar in patients with and without ERR, expect ERR was observed more frequently among patients who received TPF and GP regimen as first‐line chemotherapy. The median OS was 34.3 months for ERR patients vs 22.2 months for non‐ERR (*P* < .001, Figure 1A). The median PFS for ERR patients was significantly longer than that for non‐ERR (10.2 vs 7.4 months, respectively, *P* < .001, Figure 1B). The ERR predicted PFS and OS with a higher accuracy (AUC = 0.61 and 0.58). Subgroup analysis was conducted to investigate the benefits of ERR for patients with different baseline characteristics and chemotherapy regimen. In all subgroups, there was a trend toward better OS (Figure 2) and PFS (Figure 3) in the ERR arm.

Multivariate analysis was performed on the cohort to adjust for various prognostic factors. This multivariate analysis confirmed that ERR was an independent prognostic factor associated with OS (HR = 0.591, 95% CI, 0.495‐0.705, *P* < .001) and PFS (HR = 0.586, 95% CI, 0.500‐0.686, *P* < .001), respectively. The results of the multivariate analysis are listed in Table 2. A full‐risk model combining all significant clinical variables predicted the PFS and OS with highest accuracy (AUC = 0.74 and 0.69).

**Table 2:** Multivariable analysis of the early (A) and the best radiological response (B)

Variable	Cox multivariable analysis for OS (A)			Cox multivariable analysis for PFS (A)			Cox multivariable analysis for OS (B)			Cox multivariable analysis for PFS (B)		
	HR	95% Cl	*P*	HR	95% Cl	*P*	HR	95% Cl	*P*	HR	95% Cl	*P*
tumor response (Nonresponse vs Response)	0.591	0.495 to 0.705	<.001	0.586	0.500 to 0.686	<.001	0.591	0.490 to 0.713	<.001	0.461	0.390 to 0.546	<.001
Chemotherapy regimen			.060			<.001			.044			<.001
PF	Ref.			Ref.			Ref.			Ref.		
TP	0.770	0.612 to 0.969	.026	0.746	0.611 to 0.911	.004	0.773	0.616 to 0.970	.027	0.745	0.610 to 0.910	.004
TPF	0.757	0.586 to 0.962	.023	0.574	0.464 to 0.711	<.001	0.740	0.583 to 0.938	.013	0.600	0.485 to 0.744	<.001
GP	0.884	0.681 to 1.148	.356	0.735	0.586 to 0.921	.007	0.859	0.664 to 1.112	.250	0.750	0.598 to 0.941	.013
Sex (Male vs Female)	0.817	0.638 to 1.047	.110	0.816	0.661 to 1.006	.057	0.824	0.644 to 1.056	.126	0.811	0.658 to 0.998	.048
KPS	0.975	0.945 to 1.006	.114	0.989	0.960 to 1.020	.489	0.972	0.942 to 1.003	.079	0.988	0.958 to 1.018	.415
Number of metastatic organs (One vs Two or more)	1.250	1.030 to 1.518	.024	1.229	1.037 to 1.457	.017	1.219	1.004 to 1.480	.046	1.213	1.023 to 1.439	.026
Liver metastasis (Yes vs No)	1.510	1.253 to 1.820	.001	1.555	1.322 to 1.829	<.001	1.520	1.262 to 1.831	<.001	1.559	1.326 to 1.833	<.001
Bone metastasis (Yes vs No)	1.125	0.936 to 1.351	.209	0.750	0.639 to 0.880	<.001	1.109	0.923 to 1.332	.268	0.725	0.617 to 0.852	<.001
EBV DNA level (≤1000 copies vs >1000 copies)	1.452	1.132 to 1.863	.003	1.191	0.958 to 1.481	.116	1.524	1.187 to 1.956	.001	1.187	0.963 to 1.463	.108
LDH level (≤250 vs >250)	1.417	1.165 to 1.723	.005	1.289	1.082 to 1.535	.004	1.379	1.134 to 1.677	.001	1.282	1.077 to 1.527	.005
ALP level (≤125 vs >125)	0.960	0.756 to 1.220	.739	0.869	0.702 to 1.076	.197	0.993	0.782 to 1.261	.955	1.016	0.819 to 1.261	.886
CRP level (≤3.0 vs >30.)	1.335	1.093 to 1.630	.005	1.297	1.092 to 1.539	.003	1.336	1.094 to 1.630	.004	1.312	1.105 to 1.558	.002

Abbreviations: 95% CI, 95% confidence interval; GP, gemcitabine and platinum. HR, hazard ratio; OS, overall survival; PFS, progression‐free survival; PF, platinum and 5‐fluorouracil; TP, docetaxel or paclitaxel and platinum; TPF, docetaxel or paclitaxel, platinum and 5‐fluorouracil;.

### 3.3 BRR and clinical outcome

Totally 533 patients achieved the BRR. Patients with the BRR also had significantly longer OS (31.4 vs 20.9 months; HR, 0.597; 95% CI, 0.497‐0.717; *P* < .001, Appendix Figure 1A) and PFS (10.4 vs 6.5 months; HR, 0.442; 95% CI, 0.374‐0.521; *P* < .001, Appendix Figure 1B) than those without in univariable analysis (Appendix Table 1). The BRR predicted PFS and OS with a higher accuracy (AUC = 0.61 and 0.59). Multivariate analysis was performed on the cohort to adjust for various prognostic factors. This multivariate analysis confirmed that BRR was an independent prognostic factor associated with OS (HR = 0.591, 95% CI, 0.490‐0.713, *P* < .001) and PFS (HR = 0.461, 95% CI, 0.390‐0.546, *P* < .001), respectively. The results of the multivariate analysis are listed in Table 2.

### 3.4 Correlation between the ERR and BRR

Among patients with the BRR, only 78 (14.6%) did not achieve ERR. The highest kappa agreement (r = .73) and Pearson correlation coefficient (r = .74) were seen between the ERR and BRR. Furthermore, patients achieving an ERR were 5.8 times more likely to have a BRR. ERR had sensitivity of 85.4% and specificity of 89.3% for detection of a BRR. The positive predictive value (PPV) and negative predictive value (NPV) for this model were 72.7% and 96.8%, respectively.

### 3.5 Analyses of predefined response categories

**Figure 4:**
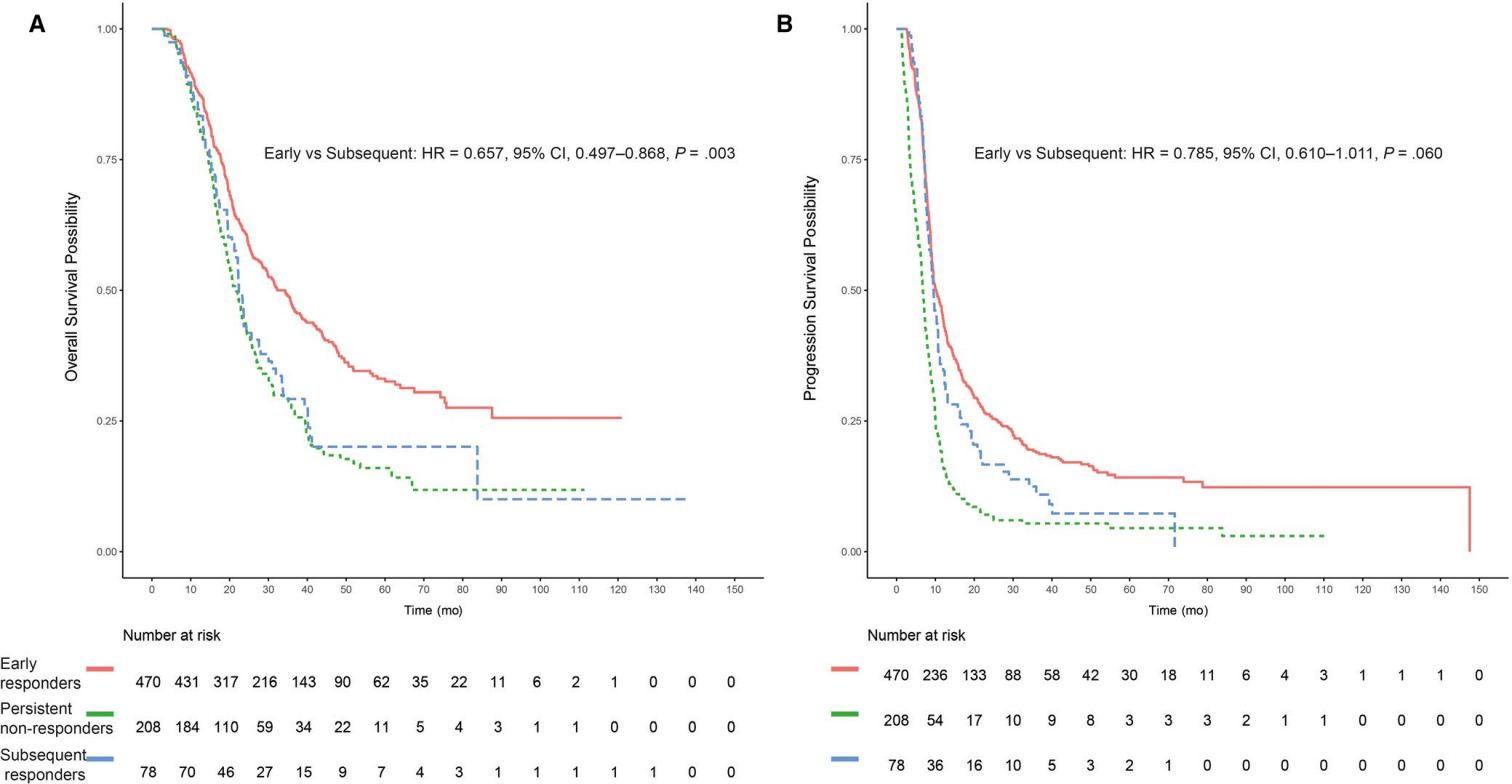
Kaplan‐Meier curves generated among entire population to compare survival. (A) Overall survival (B) Progression‐free survival

Of the 286 patients who had non‐ERR, 78 (27.3%) patients who achieved CR or PR as the best response after repeated chemotherapy were defined as the subsequent responders, and 208 (72.7%) patients were maintained nonresponse despite repeated chemotherapy, who were defined as the persistent nonresponders. According to the predefined response categories, three survival curves from ERR patients, subsequent responders, and persistent nonresponders were significantly different, with median OS times of 34.3, 22.4, and 21.7 months (*P* < .001, Figure 4) and median PFS times of 10.2, 9.5, and 6.7 months, respectively (*P* < .001, Figure 4). Consistent with the univariate results, multivariate analysis revealed that the ERR was associated with significantly improved OS (HR, 0.657; 95% CI, 0.496‐0.871; *P* = .003), but not associated with significantly improved PFS (HR, 0.855; 95% CI, 0.661‐1.097; *P* = .233) compared with subsequent responders.

## 4 DISCUSSION

This retrospective study was based on a large cohort of consecutive patients with newly diagnosed mNPC who received first‐line platinum‐containing chemotherapy. There are several notable findings from this study. Firstly, our data showed that ERR has prognostic value in terms of PFS and OS for mNPC. Secondly, there is a correlation between ERR and BRR. Finally, ERR may be a more sensitive predictor to assess the response to systemic treatment than BRR for mNPC patients.

The correlation between radiological response and survival has been shown in different types of tumors such as metastatic CRC, hepatocellular carcinoma, and breast cancer.^8^, ^17^, ^18^ In accordance with previous works on other malignancies, studies have confirmed that radiological response after induction chemotherapy (IC) is correlated with clinical outcome in nonmetastatic NPC. Liu et al identified that the 3‐year PFS and 3‐year local recurrence‐free survival of patients with a satisfactory radiological response after IC were significantly better than those without.^19^ Recently, Peng et al performed an analysis of 231 nonmetastatic NPC patients treated with the TPF regimen IC.^20^ These results also showed that CR/PR after IC was associated with prolonged survival. There are few studies to explore the prognostic value of radiological response to first‐line chemotherapy in mNPC. In a retrospective analysis, 4/74 (5.4%) achieved CR, 48/74 (64.8%) PR, 12/74 (16.2%) SD, and 10/74 (13.5%) PD.^21^ Study identified significantly improved survival in liver metastases NPC patients who achieved CR or PR after chemotherapy was administered. In a recent study by Shen et al, patients with mNPC who achieved a CR during the courses of treatment were shown to have better OS than those with a noncomplete response.^22^ However, these studies were limited by their small cohorts and specific patient populations.

To our knowledge, BRR has been widely used for assessments and reporting of tumor response to treatment with antitumor agents, and a good correlation was found between OS or PFS with BRR.^8^, ^16^ However, BRR is defined as “best response” regardless of the time when it is achieved, which could take a substantial period of time to assess. Early response is assessed for each patient at the first postbaseline scan, at about 6 weeks, and has recently come to the attention of clinicians as a favorable prognostic marker for both PFS and OS in patients with other malignant cancer receiving first‐line treatment.^12^, ^13^, ^15^ Viktor et al found that those with early tumor shrinkage to chemotherapy had significantly longer OS (*P* < .0001; median: 28.5 vs 16.0 months) and PFS (*P* < .0001; median: 10.5 vs 5.3 months) compared to patients without early tumor shrinkage.^15^ Early tumor shrinkage at first posttreatment assessment served as a putative early end point in patients with metastatic RCC. Recently, Ma et al reported that early metabolic response and EBV DNA clearance could predict survival and subsequent response in NPC patients with advanced or recurrent disease.^23^ Here, our analyses firstly investigated the prognostic value of ERR compared with non‐ERR based on a large cohort of consecutive patients with mNPC. In addition, we revealed that mNPC patients with ERR exhibited significantly longer median OS, compared to nonresponders. Multivariate analysis reinforced this finding and the analysis showed that ERR was able to predict longer survival. ERR may be used as an early indicator of sensitivity to treatment and may provide clinical investigators a clinical tool to guide early on‐treatment decisions including continuation or discontinuation of therapy. An end point in clinical trials that provides an earlier indication of treatment efficacy than OR by RECIST would help save resources and accelerate the initiation of the regulatory approval process.

Our findings indicated that ERR has a strong correlation with BRR. The majority of cases with BRR were predicted by ERR. That is, patients without ERR had 72.7% possibility of response failure during repeat chemotherapy. Furthermore, we examined the tumor response of repeating chemotherapy and found that patients with ERR could survive longer than subsequent responders (patients achieved CR or PR after repeat chemotherapy). Besides, our study showed that although subsequent responders demonstrated short‐term efficacy with relatively high PFS, it failed to improve OS in these patients. Based on our findings, ERR may be a stronger prognosticator of prolonged survival in mNPC patients compared with BRR. For the patients without ERR after 6 weeks of chemotherapy, intensification of treatment, such as a combination of new antitumor drug to enhance the chemotherapy‐sensitizing effect, administration of an additional target agent, or a change of chemotherapy regimen, may be required.

There are some limitations to this study. First, this was a retrospective analysis, and the results may have been subjected to residual confounding variables. Additionally, the large dataset used in the current analysis included patients who received different chemotherapy regimens. To minimizing the impact of the different chemotherapy regimens confounder, subgroup analysis was conducted to investigate the benefits of ERR, and there was a trend toward better OS and PFS in the ERR arm. The results should be validated in a prospective trial. Finally, many researchers have suggested that it might be better to monitor treatment effects via PET/CT after, for example, two or more cycles of treatment. It is a limitation that we do not have complete PET/CT data for this analysis.

## 5 CONCLUSIONS

ERR is an independent prognostic factor in determining survival in mNPC patients who received first‐line platinum‐based chemotherapy, which may be a more sensitive predictor than BRR to evaluate the response of chemotherapy in mNPC. Validation studies are needed.

## CONFLICT OF INTEREST

The authors have declared no conflict of interest.

## AUTHOR CONTRIBUTIONS

Conception and design: Wei‐Xiong Xia, Yan‐Qun xiang, Guo‐Ying Liu; Provision of study material or patients: Yan‐Qun xiang; Data collection and analysis: Guo‐Ying Liu, Wei‐Xiong Xia, Xing Lv, Liang‐Ru Ke, Hu Liang, Wang‐zhong Li, Kui‐Yuan Liu, Shu‐Hui Lv; Data analysis and interpretation: Guo‐Ying Liu; Contribution of financial support: Yan‐Qun xiang, Wei‐Xiong Xia, Xiang Guo, Xing Lv; Manuscript writing: Guo‐Ying Liu, Wang‐zhong Li, Kang‐Qiang Peng. Response assessment: Liang‐Ru Ke, De‐Ling Wang.

## Data Availability

The datasets generated and/or analyzed during the current study are publicly available in the SYSUCC. The key raw data have been deposited into the Research Data Deposit (http://www.researchdata.org.cn)
