# Supplementary figures and images for: Prognostic value of early radiological response to first‐line platinum‐containing chemotherapy in patients with metastatic nasopharyngeal carcinoma
Source: Cancer Med. 2019 Dec 13;9(3):920–30. doi: 10.1002/cam4.2751 (PMC6997054; doi:10.1002/cam4.2751)

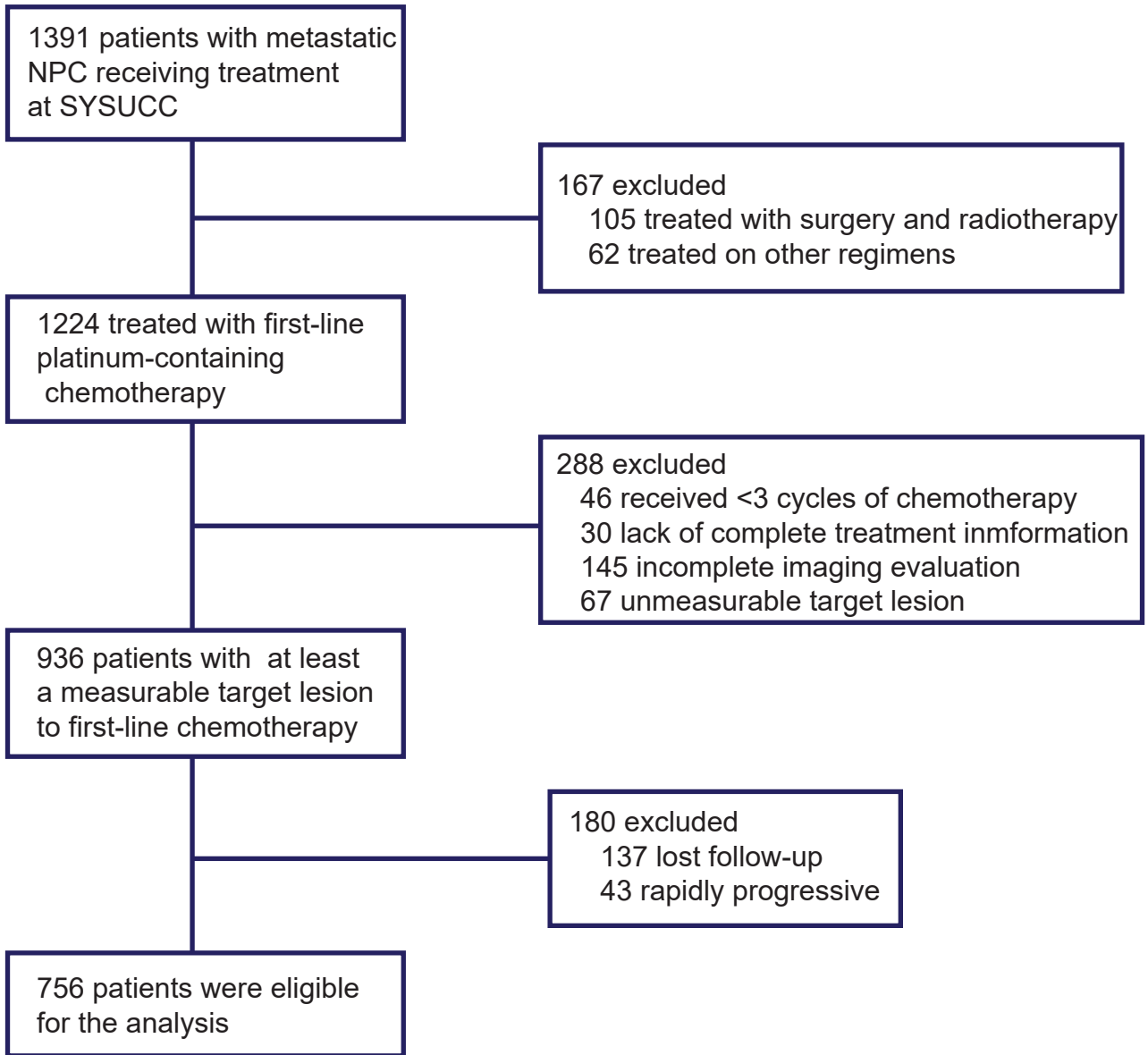

Supplement: Supplementary file 1 [file CAM4-9-920-s001.pdf]

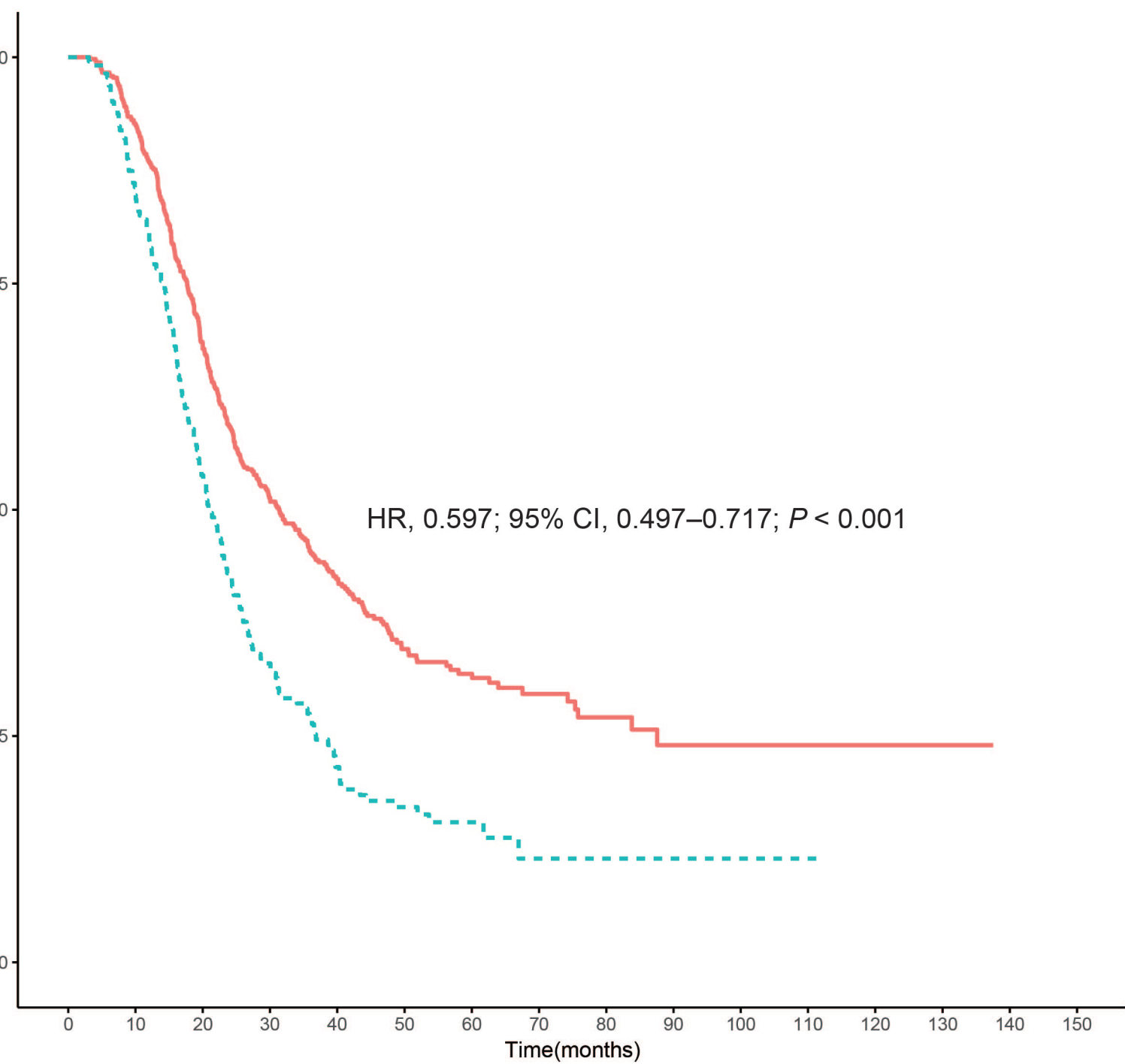

Number at risk

|     |     |     |     |     |    |    |    |    |    |   |   |   |   |   |   |
|-----|-----|-----|-----|-----|----|----|----|----|----|---|---|---|---|---|---|
| 533 | 494 | 357 | 240 | 157 | 99 | 69 | 39 | 25 | 12 | 7 | 3 | 2 | 1 | 0 | 0 |
| 223 | 191 | 116 | 62  | 35  | 22 | 11 | 5  | 4  | 3  | 1 | 1 | 0 | 0 | 0 | 0 |

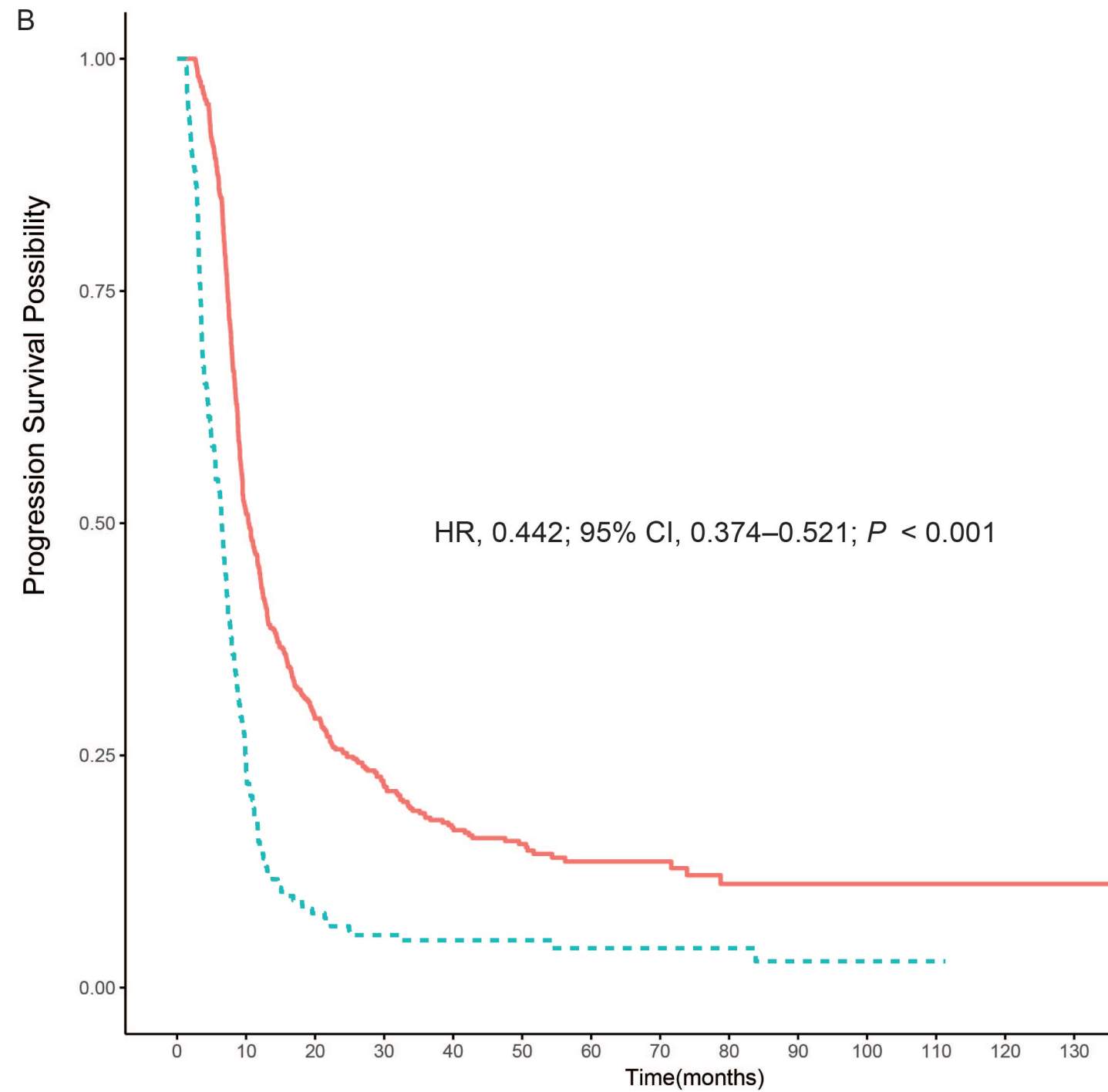

Number at risk

|     |     |     |    |    |    |    |    |    |   |   |   |   |   |
|-----|-----|-----|----|----|----|----|----|----|---|---|---|---|---|
| 533 | 272 | 149 | 98 | 63 | 45 | 32 | 19 | 11 | 6 | 4 | 3 | 1 | 1 |
| 223 | 54  | 17  | 10 | 9  | 8  | 3  | 3  | 3  | 2 | 1 | 1 | 0 | 0 |

Supplement: Supplementary file 2 [file CAM4-9-920-s002.pdf]
