# Supplementary material for: Prognostic value of early radiological response to first‐line platinum‐containing chemotherapy in patients with metastatic nasopharyngeal carcinoma
Source: Cancer Med. 2019 Dec 13;9(3):920–30. doi: 10.1002/cam4.2751 (PMC6997054; doi:10.1002/cam4.2751)
Supplement: Supplementary file 3 [file CAM4-9-920-s003.docx]

Supplemental Table 1. Univariate analysis for overall survival.

| Variable | OS | | |
| --- | --- | --- | --- |
|  | *HR* | *95% Cl* | *P* |
| Early response  (Response vs. Non-response) | 0.609 | 0.511 to 0.727 | <0.001 |
| Best response  (Response vs. Non-response) | 0.597 | 0.497 to 0.717 | <0.001 |
| Sex | 0.763 | 0.598 to 0.974 | 0.030 |
| KPS | 0.960 | 0.931 to 0.990 | 0.009 |
| Chemotherapy regimen |  |  | 0.019 |
| PF | *Ref.* |  |  |
| TP | 0.799 | 0.637 to 1.002 | 0.0053 |
| TPF | 0.693 | 0.548 to 0.878 | 0.002 |
| GP | 0.833 | 0.645 to 1.075 | 0.160 |
| Number of involved sites  (One vs. Two or more) | 1.726 | 1.446 to 2.060 | <0.001 |
| Liver metastasis  (Yes vs. No) | 1.832 | 1.539 to 2.182 | <0.001 |
| Bone metastasis  (Yes vs. No) | 1.218 | 1.024 to 1.450 | 0.026 |
| EBV DNA level  (≤1,000 copies vs. >1,000 copies) | 1.769 | 1.388 to 2.256 | <0.001 |
| LDH level  (≤250 U/L vs. >250 U/L) | 1.844 | 1.541 to 2.207 | <0.001 |
| ALP level  (≤125 U/L vs. >125 U/L) | 1.475 | 1.183 to 1.839 | 0.001 |
| CRP level  (≤3.0 mg/L vs. >3.0 mg/L) | 1.646 | 1.365 to 1.986 | <0.001 |

Abbreviations: PF, cisplatin and 5-fluorouracil; TP, taxane and cisplatin; KPS, Karnofsky Performance Status; TPF, taxane, cisplatin and 5-fluorouracil; GP, gemcitabine and cisplatin. HR, hazard ratio; 95%*CI*, 95% confidence interval.
